# Secretome from Human Mesenchymal Stem Cells-Derived Endothelial Cells Promotes Wound Healing in a Type-2 Diabetes Mouse Model

**DOI:** 10.3390/ijms23020941

**Published:** 2022-01-15

**Authors:** Valeska Ormazabal, Estefanía Nova-Lampeti, Daniela Rojas, Felipe A. Zúñiga, Carlos Escudero, Paola Lagos, Alexa Moreno, Yanara Pavez, Camila Reyes, Milly Yáñez, Mabel Vidal, Guillermo Cabrera-Vives, Katherine Oporto, Claudio Aguayo

**Affiliations:** 1 Department of Pharmacology, Faculty of Biological Sciences, Universidad de Concepción, Concepción 4030000, Chile; vormazabal@udec.cl (V.O.); paalagos@egresados.ubiobio.cl (P.L.); 2 Department of Clinical Biochemistry and Immunology, Faculty of Pharmacy, Universidad de Concepción, Concepción 4030000, Chile; enova@udec.cl (E.N.-L.); fzuniga@udec.cl (F.A.Z.); alexamoreno1795@gmail.com (A.M.); yanara.pavez@gmail.com (Y.P.); camireyesviver@gmail.com (C.R.); katyoportopalma@gmail.com (K.O.); 3 Department of Animal Pathology, Faculty of Veterinary Sciences, Universidad de Concepción, Chillan 3787000, Chile; drojasm@udec.cl; 4 Vascular Physiology Laboratory, Department of Basic Sciences, Universidad del Bio-Bio, Chillan 3787000, Chile; cescudero@ubiobio.cl; 5 Group of Research and Innovation in Vascular Health (GRIVAS Health), Chillan 3787000, Chile; 6 Department of Pathological Anatomy, Las Higueras Hospital, Talcahuano 4030000, Chile; dramilly@gmail.com; 7 Department of Computer Science, Faculty of Engineering, Universidad de Concepción, Concepción 4030000, Chile; mabvidal@udec.cl (M.V.); guillecabrera@inf.udec.cl (G.C.-V.)

**Keywords:** human mesenchymal cells-derived endothelial cells, secretome, hyperglycemic conditions, regeneration

## Abstract

Tissue regeneration is often impaired in patients with metabolic disorders such as diabetes mellitus and obesity, exhibiting reduced wound repair and limited regeneration capacity. We and others have demonstrated that wound healing under normal metabolic conditions is potentiated by the secretome of human endothelial cell-differentiated mesenchymal stem cells (hMSC-EC). However, it is unknown whether this effect is sustained under hyperglycemic conditions. In this study, the wound healing effect of secretomes from undifferentiated human mesenchymal stem cells (hMSC) and hMSC-EC in a type-2 diabetes mouse model was analyzed. hMSC were isolated from human Wharton’s jelly and differentiated into hMSC-EC. hMSC and hMSC-EC secretomes were analyzed and their wound healing capacity in C57Bl/6J mice fed with control (CD) or high fat diet (HFD) was evaluated. Our results showed that hMSC-EC secretome enhanced endothelial cell proliferation and wound healing in vivo when compared with hMSC secretome. Five soluble proteins (angiopoietin-1, angiopoietin-2, Factor de crecimiento fibroblástico, Matrix metallopeptidase 9, and Vascular Endothelial Growth Factor) were enriched in hMSC-EC secretome in comparison to hMSC secretome. Thus, the five recombinant proteins were mixed, and their pro-healing property was evaluated in vitro and in vivo. Functional analysis demonstrated that a cocktail of these proteins enhanced the wound healing process similar to hMSC-EC secretome in HFD mice. Overall, our results show that hMSC-EC secretome or a combination of specific proteins enriched in the hMSC-EC secretome enhanced wound healing process under hyperglycemic conditions.

## 1. Introduction

In healthy individuals, wound healing involves multiple processes, including extracellular matrix remodeling, synthesis of pro-inflammatory mediators, and new vessel formation [1]. However, when the wounding healing response becomes abnormal, excessive healing or an ulcerative lesion (chronic wound) occurs. Thus, chronic wounds are defined as tissue injuries that have not been repaired on time or in the correct sequential order to recover the anatomical and functional integrity of the damaged tissue [2]. Chronic wounds are generally associated with poor cellular responses, reduced endogenous pro-healing growth factors, or impaired activity in the wounded microenvironment [3].

Diabetes mellitus (DM) is characterized by hyperglycemia and alterations in the metabolism of hydrates of carbon, fats, and proteins [4,5]. Despite that, poor glycemic control may occur, leading to metabolic and systemic complications, such as the development of foot ulcers (DFU). DFU is characterized by skin ulceration, an injury that affects the total thickness of the foot dermis.

Conventional wound management involves recovering metabolic homeostasis, ensuring adequate blood perfusion, and local wound treatment with dressings and coverage. However, there is a high number of patients who are refractory to conventional therapeutic management. In this regard, several experimental strategies have been demonstrated to accelerate the process of wound healing in diabetic patients [6]. For instance, previous studies have shown that mesenchymal stem cells (MSCs) can differentiate into several lineages to induce tissue regeneration. Furthermore, MSCs can promote angiogenesis, endothelial cell recruitment, extracellular matrix remodeling, and an anti-inflammatory environment. In addition, several studies have shown that MSC secreted factors are critical players in the wound healing process [5,6,7,8,9,10].

We have previously demonstrated that the secretome of endothelial cells derived from MSCs (hMSC-EC) accelerated wound closure in normoglycemic mice [11]. However, whether this secretome can promote in vivo wound healing in a type-2 diabetes mouse model remains unknown. Therefore, we aim to investigate the capacity of hMSC-EC secretome to improve wound healing in vivo in a type-2 diabetes model induced by a high fat diet (HFD). Our results demonstrate that hMSC-EC secretome or a combination of specific proteins enriched in the hMSC-EC secretome enhanced wound healing under hyperglycemic conditions.

## 2. Results

### 2.1. hMSC-EC Secretome Promotes Endothelial-like Cells Growth In Vitro

**Figure 1:**
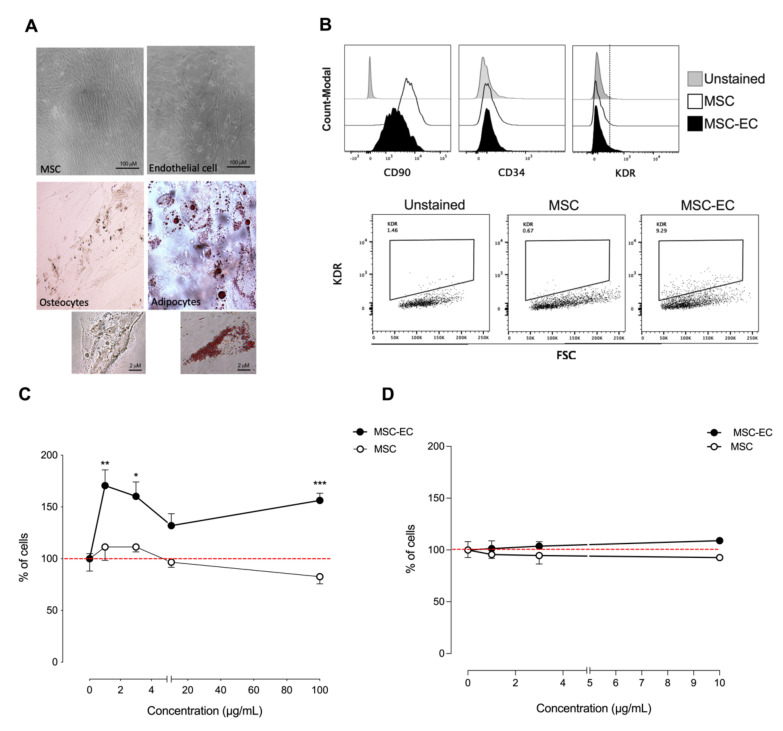
Phenotypic characterization of human endothelial cell-differentiated mesenchymal stem cells (hMSC-EC) and functional characterization of hMSC-EC-secretome in endothelial-like and keratinocytes cell lines. (**A**) hMSCs were differentiated into endothelial cells, osteocytes, and adipocytes using different differentiation media. The cells were stained for lipid droplets (Oil Red O staining) or calcium deposits (Von Kossa staining), respectively (**B**). The expression of CD90, CD34, and KDR was measured in hMSCs and hMSCs-EC after 14 days of culture using flow cytometry. hMSC and hMSC-EC secretome were added into ECV-304 and HaCaT at 1, 3, and 10 μg/mL for 24 h. The % of ECV-304 (**C**) and HaCaT (**D**) cells was calculated using the sulforhodamine B (SRB) assay. The values were expressed as a percentage of the control without secretome. The red line corresponds to the basal level. One-way ANOVA followed by a Tukey–Kramer test was used to examine the difference between experimental group. Values are expressed as mean and S.E.M. *n* = 4. Statistical significance is represented as * *p* < 0.05, ** *p* < 0.01, *** *p* < 0.005 vs. control.

The capacity of human mesenchymal stem cells (hMSCs) to differentiate into adipocytes and osteocytes was evaluated in vitro by using a specific differentiation medium (Figure 1A). Differentiation of Wharton’s jelly-derived MSCs to endothelial cells (identified as hMSC-EC in this manuscript), osteocytes, and adipocytes, was confirmed by analyzing surface markers, cell morphology, calcium deposition, and inclusion of lipid vesicles (Figure 1A). Osteogenic differentiation was confirmed with calcium mineralization by Von Kossa staining and adipocyte differentiation was confirmed with the presence of lipid droplets by oil Red-O dye.

For endothelial cell differentiation, the flow cytometry analysis confirmed the presence of CD90 and the absence of CD34 in hMSCs and hMSC-EC; however, hMSC-EC expressed reduced levels of CD90 in comparison with hMSC, suggesting that the differentiated cells begin to lose their mesenchymal phenotype (Figure 1B). In addition, the endothelial functional differentiation marker kinase insert domain receptor (KDR or VEGFR2) was analyzed. We observed that hMSCs-EC expressed higher levels of KDR than hMSC, suggesting endothelial differentiation at early-stage (Figure 1B). In addition, real-time PCR analysis of endothelial marker showed higher expression of *eNOS, CD34, CD31, Tie-2*, and KDR in hMC-EC than hMSC (Figure S1). These data confirm previous results suggesting that Wharton’s gelatin hMSC differentiation towards an endothelial phenotype is occurring.

We then evaluate the effect of secretomes on cell proliferation using endothelial-like cell line ECV-304 and keratinocyte cell line HaCaT. Secretomes of hMSC and hMSC-EC were tested at different protein concentrations for 24 h. We found that hMSC-EC secretome significantly increased the cell number of ECV-304 at 1 and 3 μg/mL compared to hMSC secretome (Figure 1C). At the same time, no significant differences were observed in keratinocyte cell line HaCaT (Figure 1D). These results suggest that hMSC-EC secretome has a soluble factor(s) that selectively promotes endothelial cell proliferation.

### 2.2. High Fat Diet-Induced Metabolic Disturbance Associated with Type-2 Diabetes in C57Bl/6J Mice

**Figure 2:**
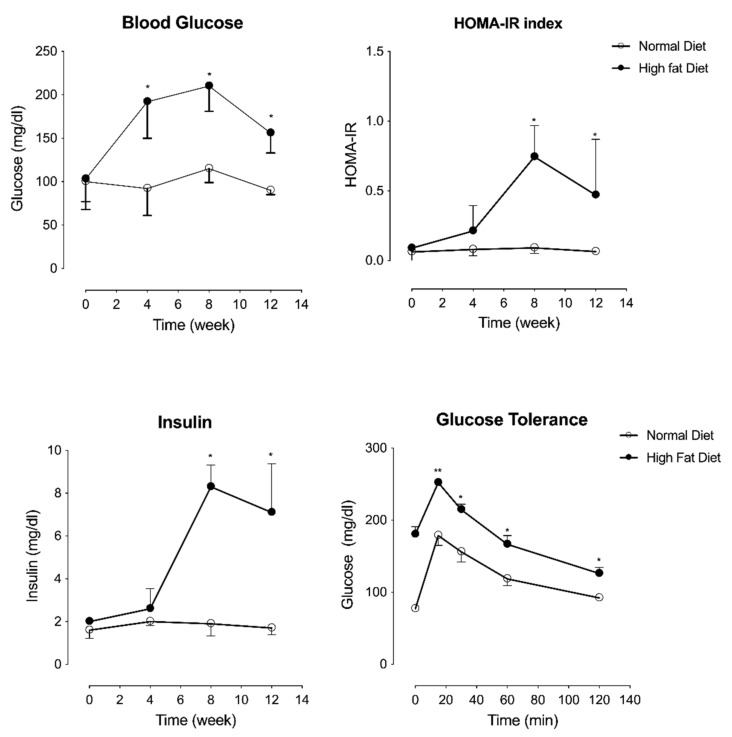
Metabolic characterization of the type-2 diabetes mouse model. Blood glucose, HOMA-IR, and insulin levels were measured in mice maintained under normal (Control Diet) or high fat diet. Glucose tolerance was measured in mice maintained under normal or high fat diet at baseline and 15, 30, 60, and 120 min after glucose administration. One-way ANOVA followed by a Tukey–Kramer test was used to examine the difference between experimental group. Values are expressed as mean, and S.E.M. *n* = 5. Statistical significance is represented as * *p* < 0.05 and ** *p* < 0.01.

To evaluate in vivo wound healing capacity of the hMSC-EC secretome in a hyperglycemic model, we used the HFD mice model. Characterization of this mice model included measurement of weight, fasting glucose levels, lipid profile, and insulin before and after 4, 8, and 10 weeks post-dietary intervention. HFD mice presented higher levels of glycemia, HOMA-IR index, and insulin in comparison with CD (Figure 2). Moreover, HFD mice exhibited increased glucose-stimulated insulin secretion at 15, 30, 60, and 120 min post-injection (Figure 2). At the same time, no significant differences were observed between HFD and CD in respect to LDL cholesterol, HDL cholesterol, and fasting triglyceride levels (Figure S2). However, when the fat mass was quantified, HFD mice accumulated 2.5 times the visceral fat than CD mice (Figure S2). Nevertheless, total antioxidant content in plasma was also evaluated in both HFD and CD mice. Lower total antioxidant content was observed in HFD mice than CD mice at 12 weeks after diet intervention (Figure S2).

### 2.3. hMSC-EC Secretome Improved the Rate of Wound Closure In Vivo

**Figure 3:**
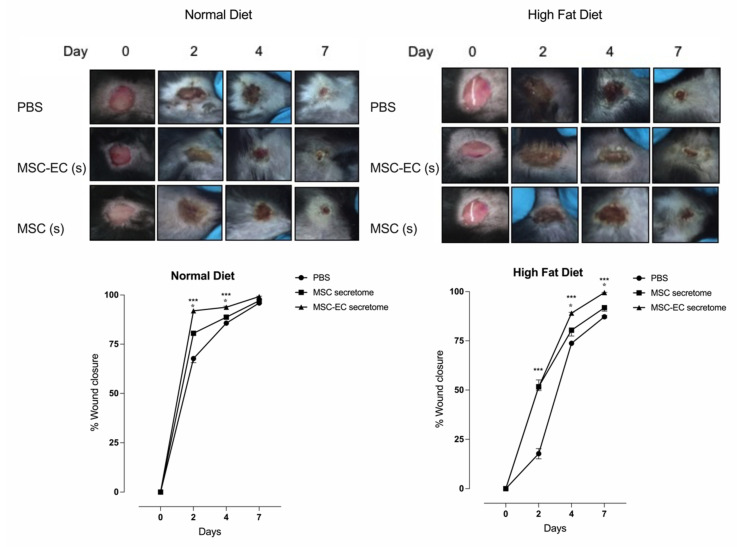
The effect of human mesenchymal stem cells (hMSCs) and human endothelial cell-differentiated mesenchymal stem cells (hMSC-EC) secretome in wound healing in a type-2 diabetes mouse model. In vivo wound healing in mice under a normal (Control Diet) or high fat diet was measured in the absence or presence of MSC or MSC-EC secretome. Images and total values of wound closure were shown at 2, 4, and 7 days post-injury. One-way ANOVA followed by a Tukey–Kramer test was used to examine the difference between experimental group. Values are expressed as mean, and S.E.M., *n* = 5. Statistical significance is represented as * *p* < 0.05 MSC-EC vs. MSC, *** *p* < 0.01 MSC-EC vs. PBS was considered significant.

After establishing the mouse model of hyperglycemia, we evaluated the capacity of hMSC or hMSC-EC secretome to stimulate wound closure in vivo. Basally, HFD mice showed a delay in wound healing compared to CD mice (Figure 3). However, complete closure of the wound was observed after nine days of dietetic intervention for both conditions.

The effect of hMSC or hMSC-EC secretome was compared in both groups of mice. hMSC-EC secretome enhanced in vivo wound healing in HFD and CD mice compared with mice treated with vehicle or hMSC secretome (Figure 3). Despite that, HFD mice treated with hMSC-EC secretome showed a delay in the wound healing process compared with CD mice treated either with hMSC or hMSC-EC secretome. Thus, while 80% and 90% of wound closure were found at 4 days after injury in the HFD group, a similar closure was found 2 days earlier in CD mice.

After completed wound healing, animals were euthanized, and pieces of skin were recovered for histological analysis. The histological results showed more significant wound healing in the histological sections from the skin of mice treated with hMSC-EC secretome than those treated with either hMSC secretome or vehicle (Figure S3).

### 2.4. Hallmark in the Proteomic Analysis of hMSC-EC Secretome

**Figure 4:**
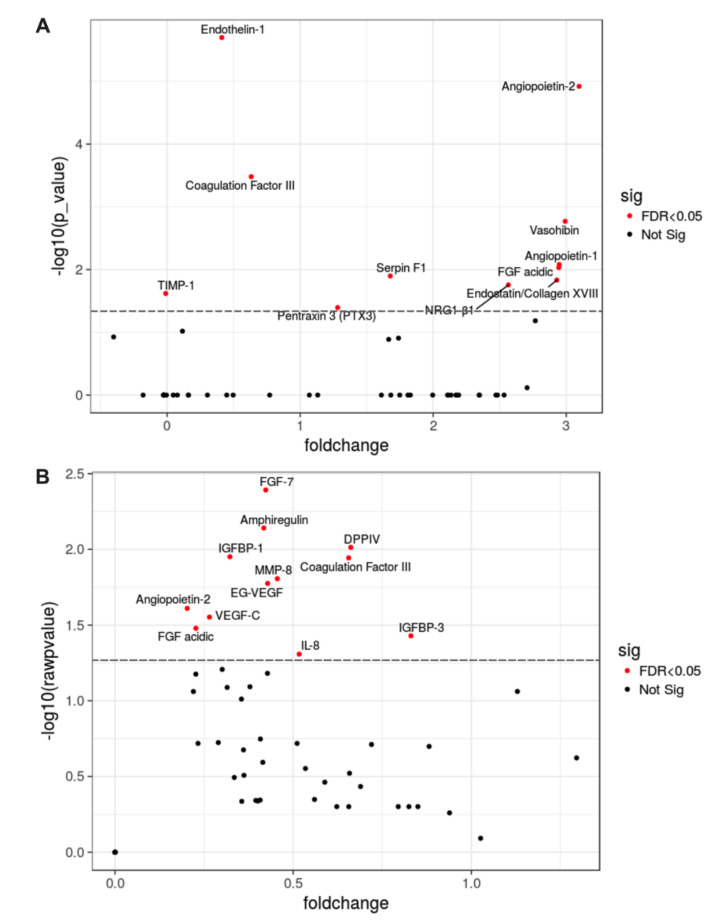
Differential proteomic profile between human mesenchymal stem cells (hMSCs) and human endothelial cell-differentiated mesenchymal stem cells (hMSC-EC). Secreted proteins from MSC and MSC-EC (1 μg/mL) we identified with a Proteome Profiler Human Array and analyzed with an (**A**) 2way ANOVA and (**B**) Welch two-sample t-test according to the mean fold changes for each protein. Volcano plots show proteins with a *p*-value < 0.05 between MSC and MSC-EC. The increased fold change for MSC-EC proteins compared to MSC proteins was considered for values > 0 (foldchange). *n* = 4.

To define the profile of the molecular mediators of angiogenesis/vasculogenesis secreted by hMSC and hMSC-EC, we analyzed their secretome by using a Proteome Profiler Human Array. We were able to identify a particular hallmark in the proteomic profile of hMSC-EC-secretome characterized by up-regulation of at least 11 proteins, including Angiopoietin-1 (Ang-1), Angiopoietin-2 (Ang-2), Coagulation Factor III, Endothelin-1, Factor de crecimiento fibroblástico 7 (FGF-7), Factor de crecimiento fibroblástico acidic (FGF acidic), Matrix metallopeptidase 8 (MMP-8), neuregulin β1 (NRG1-β1), Serpin F1, Vasohibin, and VEGF-C (Figure 4). Higher levels of FGF-7), Matrix metallopeptidase 9 (MMP-9), Vascular Endothelial Growth Factor C (VEGF-C), angiopoietin-1, and angiopoietin-2 were detected in hMSC-EC compared to hMSC; thus, these proteins were selected to evaluate further whether these factors may resemble the wound repair process observed with the whole secretome. Of note, the increment of FGF-7, MMP-9, VEGF-C, and angiopoietin-1 were similar, whereas angiopoietin-2 levels were half than the other proteins.

### 2.5. Significantly Enriched Proteins in MSC-EC Secretome Promoted Wound Healing in a Type-2 Diabetes Mouse Model

**Figure 5:**
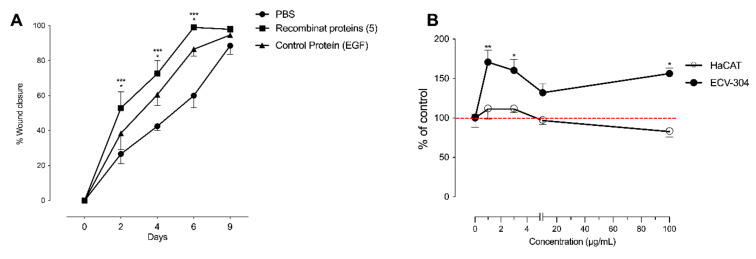
The effect of recombinant proteins differentially secreted by human endothelial cell-differentiated mesenchymal stem cells (hMSC-EC) in wound healing in a type-2 diabetes mouse model. (**A**) In vivo wound healing in mice under a high fat diet was measured in the absence or presence of recombinant proteins differentially secreted by MSC-EC (Ang-1, Ang-2, FGF-7, MMP-9, and VEGF-C) and control protein EGF. Total values of wound closure were measured at 2, 4, 7 and 9 days post-injury. (**B**) Recombinant proteins were added into ECV-304 and HaCaT cell cultures at 1, 3, 10, and 100 μg/mL for 24 h. The % of ECV-304 and HaCaT cells were calculated using the SRB assay. One-way ANOVA followed by a Tukey–Kramer test was used to examine the difference between experimental group. The values were expressed as a percentage of the control. The red line corresponds to the basal level. Values are expressed as mean, and S.E.M., *n* = 5. Statistical significance is represented as * *p* < 0.05 MSC-EC vs. MSC, *** *p* < 0.01 MSC-EC vs. PBS was considered significant. In B. * *p* < 0.05 and ** *p* < 0.01 vs. control.

Ang-1 and 2, FGF-7, MMP-9, and VEGF-C were tested in vivo and in vitro. The protein mixture was prepared at 1.0 μg/mL for FGF-7, MMP-9, VEGF-C, and Ang-1, while Ang-2 was prepared 0.5 μg/mL. As shown in Figure 5, the recombinant proteins significantly increased the speed of wound closure at days 2, 4, and 6 compared to control conditions in HFD mice (Figure 5A). As observed with the whole secretome, complete closure of the wound was observed after 9 days post intervention.

In vitro experiments showed that the cocktail of recombinant proteins (0–100 μg/mL) increased cell numbers of ECV-304, but not HaCaT (Figure 5B).

## 3. Discussion

Our results revealed that human endothelial cell-differentiated mesenchymal stem cells (hMSC-EC) secretome improved wound healing in HFD mice. Additionally, we identified a hallmark of proteins enriched in the hMSC-EC secretome. A cocktail of five recombinant growth factors improved wound healing to a similar extent to the whole secretome in HFD mice. Therefore, hMSC-EC secretome, particularly a cocktail of angiopoietin-1 and 2, FGF-7, MMP-9, and VEGF-C, are potential new therapeutic targets for improving wound-healing in diabetes.

### 3.1. Mesenchymal Stem Cells and Wound Healing

MSCs have been studied for their high potential for differentiation, proliferation, plasticity, low immunogenicity, and relatively easy culture. Those cells have been used in regenerative medicine to repair injured cutaneous structures. Our previous work demonstrated the ability of MSCs to differentiate into endothelial cells, which in turn induced angiogenesis and accelerated wound closure [11]. A series of studies confirmed the ability of MSCs to differentiate into vascular cells [12,13,14]. Previous results by our group have shown that MSC differentiated into hMSC-EC, expressing endothelial markers, such as *CD31*, *KDR*, and *eNOS*, and producing nitric oxide [11]. Others have demonstrated that specific endothelial cell phenotype and functional genes were up-regulated in MSCs stimulated under differentiating endothelial medium [15,16,17]. Therefore, our results further confirm the differentiation capacity of hMSCs to hMSC-EC. In this manuscript, we further extend these findings to analyze the potential use of hMSC-EC secretome to improve the wound healing process in HFD mice.

The analysis of the contribution of MSCs or MSCs secretome to the healing process has been extensively studied. For instance, Kato et al. (2014) showed that MSCs could enhance keratinocyte viability and promote migration. Furthermore, MSCs increased angiogenesis and decreased the level of MMP-9 in diabetic rats [18]. In addition, Wu et al. (2007) showed that injection of MSC accelerates skin regeneration and secretion of angiogenic growth factors during the wound healing process in diabetic rats [19]. Similarly, Fong et al. (2014) showed that conditioned medium harvest from MSC (MSC-CM) increased the expression of intercellular adhesion molecules and VEGF-A that favored angiogenesis, epithelialization, and collagen deposition [20]. These findings demonstrated the importance of MSCs or MSCs secretome in regulating inflammation, apoptosis, and modulating angiogenesis during the wound-healing process.

### 3.2. Paracrine Function of MSC

It is currently well-described that MSCs exert their function directly and via paracrine signaling mediated by the synthesis and release of growth factors, cytokines, and chemokines, including transforming growth factor (TGF-β), Keratinocyte Growth Factor (KGF), Effects of epidermal growth factor (EGF), Platelet-derived growth factor (PDGF), interleukin (IL), and vascular endothelial growth factor (VEGF) [20,21,22]. These factors stimulate cell adhesion at the site of injury, promoting the holding of MSC in the injured area, which then enhances the secretion of chemokines resulting in neovascularization and formation of inflammation infiltrate, containing predominantly mononuclear cells. Our data identified at least 11 angiogenic proteins up-regulated in MSC-EC secretome in comparison with MSC secretome. Those proteins included Ang-1, Ang-2, FGF-7, MMP-9, and VEGF-C. These findings agree with previous reports using MSC-CM, which identified up-regulation of IGF-1, TGF-β1, VEGF, angiogenin, and MCP-3 [23,24]. All cytokines or growth factors positively regulate several events such as osteogenesis, angiogenesis, cell migration, proliferation, and osteoblast differentiation [23,24].

Several studies reported that VEGF secretion by MSCs contributes to angiogenesis as vessel density was increased with MSCs compared with silencing VEGF-MSCs [25,26,27,28]. Kuchroo et al. showed that MMP-2, MMP-3, and MMP-9 promote angiogenesis and tube formation [29,30]. The Ang-1 and Ang-2 represent other proteins of the family of angiogenic factors [31]. Ang-1 mediates neovessel maturation into more complex and larger vascular structures and maintains vessel integrity [32]. Ang-2 is an endogenous antagonist of Tie2 that blocks Ang1-Tie2 signaling [32,33]. It has been shown that the secretome from bone marrow-derived-MSCs that promote endothelial tube formation contained VEGF and Ang-1, but not Ang-2. Unlike their results, we identified Ang-1 and Ang-2, in our proteomic array, with a different fold change. Therefore Ang-2 can promote extracellular matrix remodeling by enhancing fibroblast cell survival and secretion of the extracellular matrix components such as collagen, elastin, and fibronectin, supporting connective tissue regeneration. Such trophic factors may provide a supportive microenvironment in the damaged tissue, enhancing cell survival, renewal, and differentiation. Those phenomena modulate inflammatory reactions and induce angiogenesis, which eventually leads to the regeneration of tissue injury.

Due to the enormous potential of autocrine/paracrine factors in regenerative medicine, various cytokines, chemokines, growth factors, hormones, extracellular matrix proteins, and matrix-remodeling enzymes have been identified using proteomic techniques [34,35]. Proteomic studies have identified several soluble factors that could contribute to the regeneration process [35]. However, from the therapeutic perspective, it is feasible that a mixture of bioactive factors rather than a unique protein may be clinically relevant. Accordingly, studies in the hind limb ischemic model have reported a synergistic relationship between growth factors. The combination of factors leads to a superior therapeutic potential compared to independent factors [36,37]. Despite that, the appropriate mix of bioactive factors that recapitulate the beneficial effects observed with MSC alone represents a substantial challenge to overcome.

In terms of clinical studies, a recent review has described some disadvantages of using different MSC types in clinical and pre-clinical trials [38,39]. Whereas embryonic and pluripotent MSCs have increased malignancy risk, bone marrow, peripheral blood, umbilical cord, and adipose-derived-MSCs have limited differentiation potential, reduced cell availability influenced by the comorbidities [38] and low cell survival rates after implantation [39]. These disadvantages support the requirement of novel treatments based on MSC-secreted proteins such as conditioned media or, more recently, MSC-extracellular vesicles, including exosomes [40]. However, to date, these potential approaches have not been used in diabetic patients with chronic wound healing. This manuscript used a combination of five proteins that improve wound healing under hyperglycemic conditions, which opened the opportunity to explore their potential clinical use. Despite that, we encourage further studies to establish the best cocktail candidates based on safety, manufacturing, storage, cost, and biological effects.

## 4. Materials and Methods

### 4.1. Mice Models

All animal use and euthanasia protocols were approved by the Animal Care Committee of Universidad de Concepcion and were performed following the National Institutes of Health (NIH) guidelines. Six-week-old C57Bl/6J mice (bodyweight 20–23 g) were maintained ad libitum on water and a high fat diet (HFD) for 120 days to induce an obese and diabetic phenotype. The HFD contained high fat (35.8%), protein (23.0%), and carbohydrate (35.5%). Fat, protein, and carbohydrate provided were 58.0%, 16.4%, and 25.5%, respectively. Control mice received water and a control chow diet ad libitum for 120 days with calories provided by fat (11%), protein (23%), and carbohydrate (65%).

### 4.2. Isolation and Culture of hMSCs

Isolation and culture were performed based on previously reported [11]. Briefly, Wharton’s jelly was were digested with collagenase 10 mg/mL (37 °C for 4 h) and suspended in medium199 (M-119) (Life Technologies, Carlsbad, CA, USA) supplemented with 10% fetal bovine serum and fibroblast growth factor (50 ng/mL) (Sigma-Aldrish, St Louis, MO, USA), and cultured at 37 °C with 5% CO_2_ [11].

### 4.3. Human Mesenchymal Stem Cells (hMSCs) Differentiation

To confirm hMSCs functionality, cells were differentiated into osteocytes and adipocytes as we previously described [11]. Von Kossa and Oil Red O staining were used to confirm differentiation [29,30,31].

### 4.4. hMSCs Differentiation to Endothelial Cell (hMSC-EC)

Endothelial cell differentiation was performed as we previously described with minor modification [11]. Briefly, hMSCs were incubated in a primary culture medium (PCM) containing endothelial growth medium (Life Technologies, Carlsbad, CA, USA), fetal calf serum, and 10 mg/mL of human VEGF (GIBCO BRL Life Technologies). Characterization of endothelial-cell-like morphology and function of the hMSC-EC was confirmed as described previously [11]. 

### 4.5. Secretoma Collection

The secretome from hMSC and hMSC-EC was obtained from cells seeded in 100-mm diameter culture plates containing complete medium until 80% of confluence. The medium was then removed, and a new serum-free medium was added. After 48 h, the secretome present in the conditioned medium was concentrated using an Amicon Ultra-15 centrifugal filter (5000× *g* 1 h) (Millipore, Billerica, MA, USA) with a size cut-off of 3 kDa and used for experiments or secretome characterization (see below).

### 4.6. Cell Density/Cell Proliferation Assays Using Secretome of hMSC-EC

ECV-304 endothelial cells were grown in M-199 medium supplemented with 10% newborn calf serum, penicillin 100 U/mL, and streptomycin 100 μg/mL, at 37 °C in 5% CO_2_ [32]. The human keratinocyte cell line HaCaT was cultured in Dulbecco’s modified Eagle’s medium (DMEM) supplemented with 10% fetal bovine serum (FBS) and antibiotics (100 U/mL penicillin and 100 μg/mL streptomycin) (Life Technologies, Carlsbad, CA, USA). Cells were cultured to ~80% confluence and then used for experiments. For the cytotoxicity analysis in both cell lines, the secretome was added at 1, 3, and 10 μg/mL for 24 h. Cell proliferation was then assessed using the sulforhodamine B (SRB) assay (Roche Diagnostics, Indianapolis, IN, USA). A spectrophotometer was used to measure the optical densities of the solutions at a 560-nm wavelength.

### 4.7. Protein Analysis by Protein Array

The manufacturer’s instructions analyzed the Secretome with the Proteome Profiler^TM^ Human Angiogenesis Array Kit (R&D System, Minneapolis, MN, USA). Quantification of secretome-containing proteins was performed via standard densitometry using Quantity One Software 4.4 (Bio-Rad, Hercules, CA, USA). Differential expression analysis was performed using the R programming environment (version 3.4.4). The dataset contained 55 different capture antibodies and two donors by cells (hMSC and hMSC-EC). The log2 and mean for each protein per group and the fold change between the two groups were calculated. For protein analysis, 0.5 μg/mL of total protein was used.

### 4.8. Wound Healing Model and hMSC Transplantation

Mice feed with a normal or high fat diet was divided into four groups, and the excisional wound-splinting model was generated as described previously [11]. In brief, after anesthesia, two 6-mm full-thickness excisional skin wounds were created on each side of the midline. Each wound received 60 µL of secretome (see below), and 20 µL of growth factor-reduced Matrigel (BD Biosciences, Franklin Lakes, NJ, USA) was applied onto the wound bed. The control group only received buffer phosphate (PBS 1X, pH, 7.4) after cell injection [11]. Digital photographs of the wounds were taken at days 0, 3, 7, and 9 after injury, and the percentage of wound closure was calculated using digital measurement of the wounded area (Olympus, Tokyo, Japan). After that, biopsies of skin samples were obtained for further use in histological analysis [11].

For in vivo experiments, the FGF-7, MMP-9, VEGF-C, angiopoietin-1 and angiopoietin-2 were purchased from Sigma-Aldrish (St Louis, MO, USA). The protein mixture we prepared for future experiments emulated similar relative distribution (1.0–100 μg/mL FGF-7, MMP-9, VEGF-C, and angiopoietin-1; and half concentration for angiopoietin-2).

### 4.9. Metabolic Analysis

Blood lipid, including serum levels of total cholesterol, low-density lipoprotein (LDL), high-density lipoprotein (HDL), and triglyceride, were determined by Hitachi biochemical instrument. Additionally, blood glucose and insulin levels were determined using the AccuTrend (Roche Biochemicals, GmbH, Mannheim, Germany) and Luminex (R&D System, Minneapolis, MN, USA) sensor described by the manufacturers. Plasma total antioxidant capacity (TAC) was examined using the ferric reducing antioxidant power method [11,34].

### 4.10. Histologic Examination

Tissue samples were fixed with paraformaldehyde (3%), embedded in paraffin, cut into six-micron thick sections, and stained with hematoxylin and eosin for light microscopy. A histological score per slide was given, ranging from 1 to 10 according to the following parameters: re-epithelialization, regeneration, and angiogenesis as we described [11]. The number and area of vasculature were assessed morphometrically by examining five fields per section of the wound using Image pro plus software.

### 4.11. Statistical Analysis

Data were analyzed using standard statistical software (SPSS version 25) and GraphPad Prism 9.0. Values are expressed as mean, and S.E.M. Statistical data analysis was performed with a one-way ANOVA followed by a Tukey–Kramer. For the secretome analysis two way, ANOVA, and t-test function was used to calculate the difference per protein between hMSC and hMSC-EC. Differences were considered significant when *p*-value < 0.05.

## 5. Conclusions

This study demonstrates that hMSC-EC secretome promotes wound healing in hyperglycemic mice. We also demonstrate that a selected cocktail of five soluble factors resembles the effect of hMSC-EC secretome, making them feasible therapeutic potential candidates. Therefore, the administration of factors derived from hMSC-EC generated under strict-controlled conditions may represent a novel therapeutic approach to treat chronic wounds in diabetic patients.

## Data Availability

Data are available upon request. Contact Claudio Aguayo.

## Supplementary Materials

Supplementary materials can be found at https://www.mdpi.com/article/10.3390/ijms23020941/s1.
