# Supplementary material for: Secretome from Human Mesenchymal Stem Cells-Derived Endothelial Cells Promotes Wound Healing in a Type-2 Diabetes Mouse Model
Source: Int J Mol Sci. 2022 Jan 15;23(2):941. doi: 10.3390/ijms23020941 (PMC8779848; doi:10.3390/ijms23020941)
Supplement: Supplementary file 1 [file ijms-23-00941-s001.zip › ijms-1402692-supplementary.pdf]

International Journal of
Molecular Sciences

MDPI

Article

Secretome from Human Mesenchymal Stem Cells-Derived Endothelial Cells Promotes Wound Healing in a Type-2 Diabetes Mouse Model

Valeska Ormazabal [1], Estefanía Nova-Lampeti [2], Daniela Rojas [3], Felipe A. Zúñiga [2], Carlos Escudero [4,5], Paola Lagos [1], Alexa Moreno [2], Yanara Pavez [2], Camila Reyes [2], Milly Yáñez [6], Mabel Vidal [7], Guillermo Cabrera-Vives [7], Katherine Oporto [2] and Claudio Aguayo *,[2,5]

[1] Department of Pharmacology, Faculty of Biological Sciences, Universidad de Concepción, 4030000 Concepción, Chile; vormazabal@udec.cl (V.O.)
[2] Department of Clinical Biochemistry and Immunology, Faculty of Pharmacy, Universidad de Concepción, 4030000 Concepción, Chile; enova@udec.cl (E.N.-L.); fzuniga@udec.cl (F.A.Z.); alexamoreno1795@gmail.com (A.M.); yanara.pavez@gmail.com (Y.P.); camireyesviver@gmail.com (C.R.); katyoportopalma@gmail.com (K.O.); paalagos@egresados.ubiobio.cl (P.L.)
[3] Department of Animal Pathology, Faculty of Veterinary Sciences, University of Concepcion, 3787000 Chillan, Chile; drojasm@udec.cl (D.R.)
[4] Vascular Physiology Laboratory, Department of Basic Sciences, Universidad del Bio-Bio, 3787000 Chillan, Chile; cescudero@ubiobio.cl
[5] Group of Research and Innovation in Vascular Health (GRIVAS Health) 3787000 Chillan, Chile
[6] Department of Pathological Anatomy, Las Higueras Hospital, 4030000 Talcahuano, Chile; dramilly@gmail.com (M.Y.)
[7] Department of Computer Science, Faculty of Engineering, Universidad de Concepción, 4030000 Concepción, Chile;
mabvidal@udec.cl (M.V.); guillecabrera@inf.udec.cl (G.C.-V.)
* Correspondence: caguayo@udec.cl (C.A.); Tel.: +56-41-2207196

Methodology

Quantitative PCR

Total RNA was isolated using the Trizol Reagent (Invitrogen, Carlsbad, CA, USA) according to the manufacturers instructions. RNA quality and integrity and quality were insured by gel visualization and spectrophotometric analysis ($OD_{260/280}$). 1 μg of total RNA was reversed transcribed into cDNA for 1 hour at 37°C as described elsewhere.

mRNA for CD31, KDR, eNOS, CD34 and Tie-2 were assessed by quantitative real time PCR (qPCR) in a Rotorgene 2000 thermal cycler (Corbett Research). Thus, reactions in 25 μL were carried out using Brilliant II SYBR Green QPCR Master Mix (Agilent Technologies, USA) with 0.3 μM primers according to the manufacturer´s instruction. Primers used are described in Table 1. Samples were incubated for 4 minutes at 95°C, followed by 25 cycles of 30 seconds at 95°C, 30 seconds at 54ºC (for eNOS and CD34), 58ºC (Tie-2) or 60ºC (CD31 and KDR), 30 seconds at 72°C, and finally 7 minutes at 72°C. A single melting point was observed for all samples. The gene expression was quantified using the 2−ΔΔCt (threshold cycle) method. Thus, a delta C(T) (ΔCt) value was obtained by subtracting the cyclophilin-CT value from the CT value of the studied gene. The ΔCt mean value obtained from the control group of each gene was used to calculate the 2(-Delta Delta C(t) (ΔΔCt) of the respective gene (2−ΔΔCt). Expected size products were separated by electrophoresis on 1.5% agarose gels and visualized with ethidium bromide under UV light.

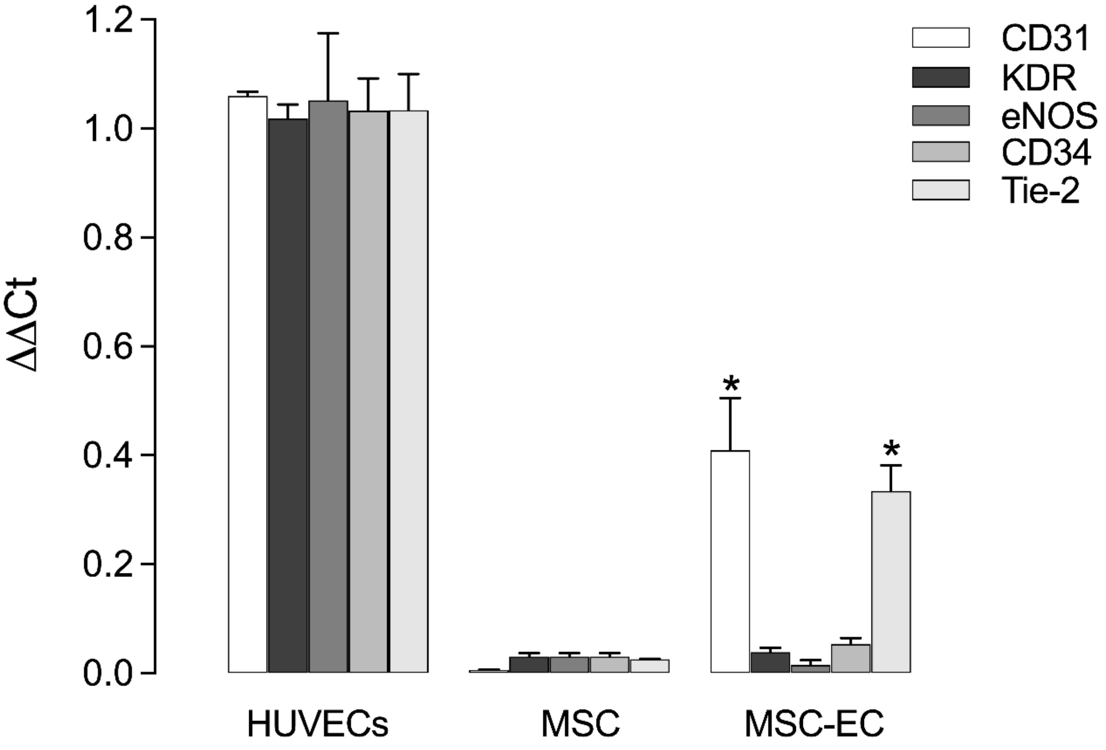

Figure S1 Expression of the endothelial marker in MSC isolated of Wharton Jelly and differentiated to MSC-EC. The mRNA levels were determined by real time-PCR for MSC and hMSC-EC. With cDNA obtained from cells, real-time PCR was performed using specific primers for *CD31, CD34, KDR, eNOS, and Tie-2*. cDNA from HUVEC was used as positive control. Statistical significance is represented as * $P < 0.05$ vs MSC.

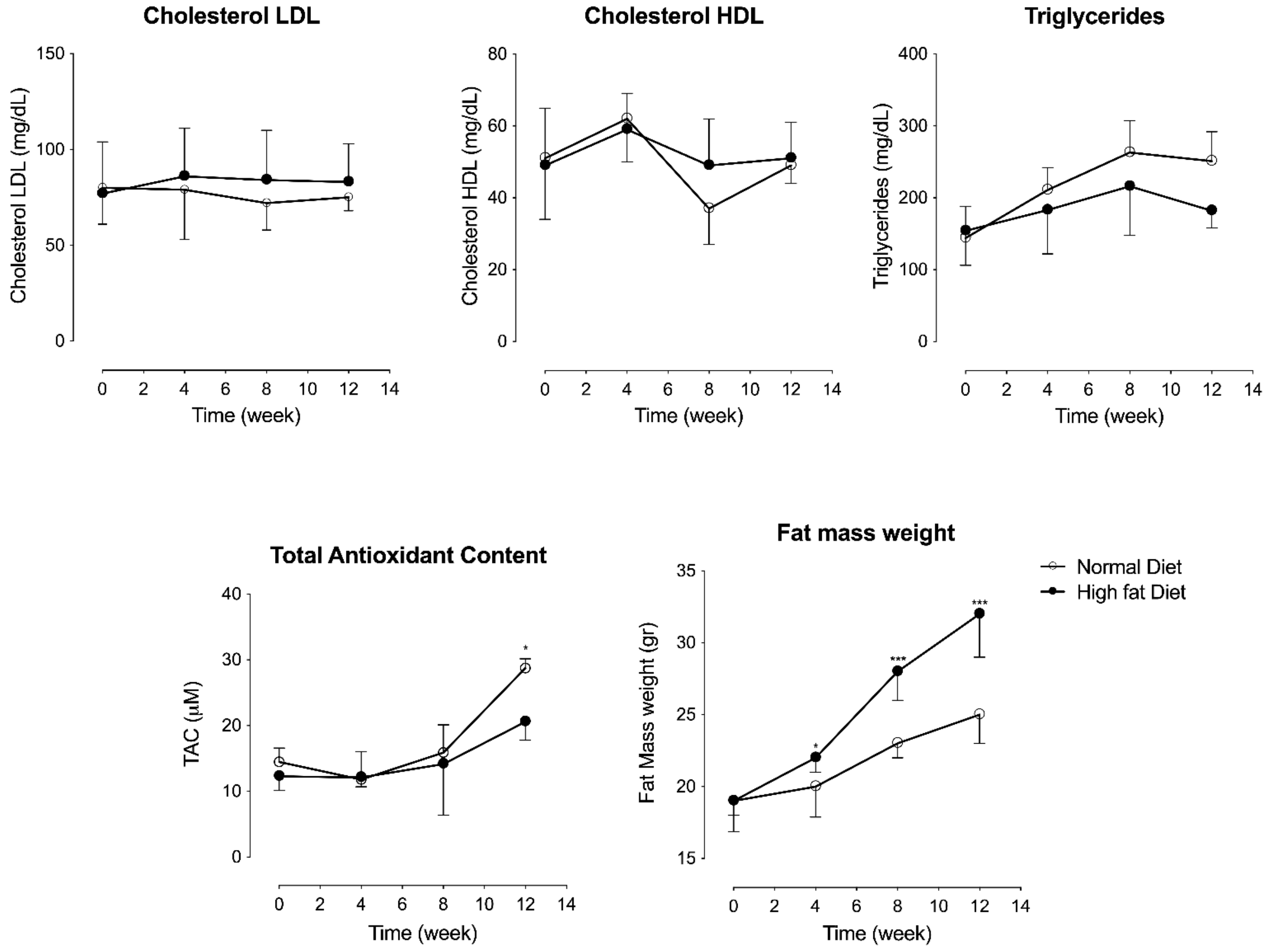

Figure S2: Metabolic characterization of the type-2 diabetes mouse model. (A) Lipidic profile and fat mass weight were measured in mice maintained under normal or high fat diet. (B) Total antioxidant content was measured in mice maintained under normal or high fat diet. Statistical significance is represented as * $P < 0.05$ and *** $P < 0.005$ vs normal diet.

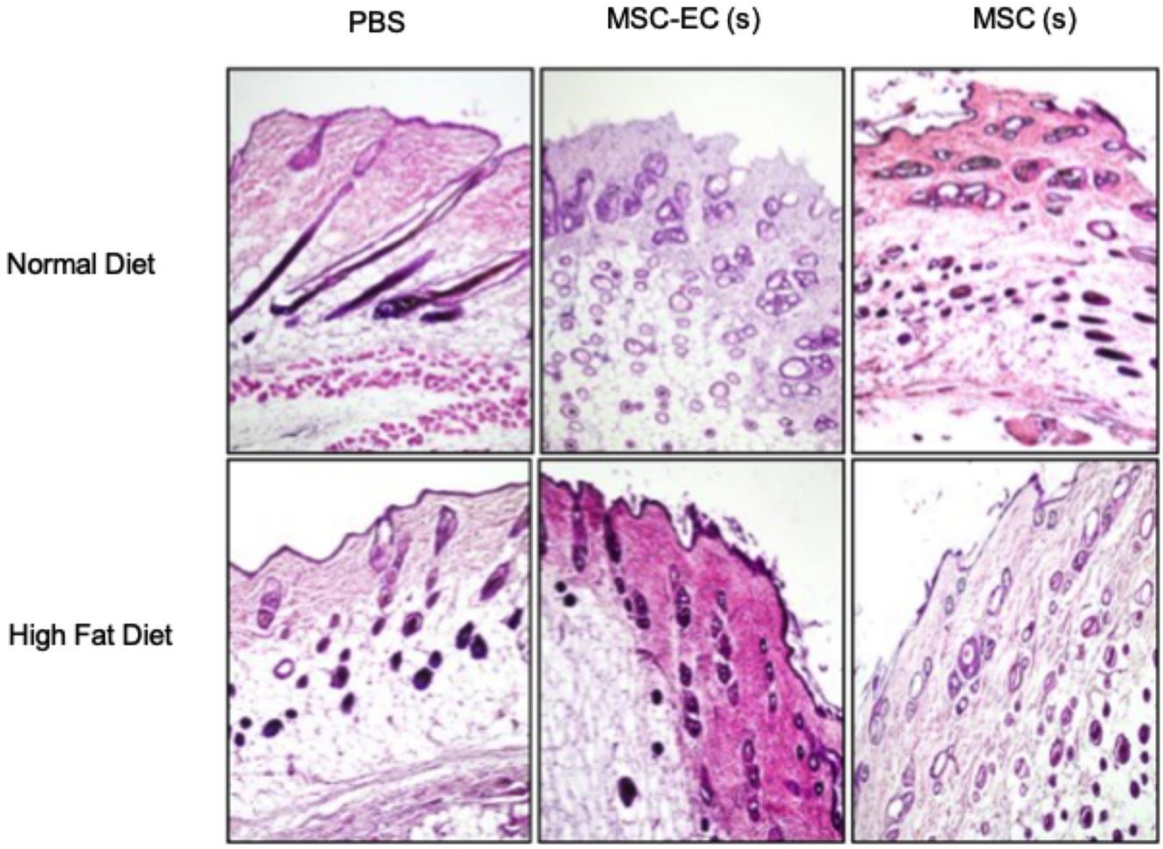

Figure S3: Histologic analysis of wounds in the wound-healing model. There were representative images of wounds (hematoxylin/eosin staining) 12 days after injury and subcutaneous injection of PBS, MSC-EC, and hWMSC in the normal and high fat diet.
